# Iranian adaptation of the Epistemic Trust, Mistrust, and Credulity Questionnaire (ETMCQ): Validity, reliability, discriminant ability, and sex invariance

**DOI:** 10.1002/brb3.3455

**Published:** 2024-03-07

**Authors:** Ahmad Asgarizadeh, Saeed Ghanbari

**Affiliations:** ^1^ Faculty of Education and Psychology Shahid Beheshti University Tehran Iran

**Keywords:** borderline personality disorder, epistemic trust, Epistemic Trust, Mistrust, and Credulity Questionnaire, mentalizing

## Abstract

**Introduction:**

Epistemic trust, or trust in transmitted knowledge, has been proposed as a critical factor in psychopathology and psychotherapy. This study aimed at evaluating the psychometric properties of the Epistemic Trust, Mistrust, and Credulity Questionnaire (ETMCQ) in Iran.

**Method:**

Data were collected from 906 participants. Along with the ETMCQ, measures of mentalizing, mindfulness, perspective‐taking, attachment, emotion dysregulation, and borderline personality disorder were administered. Confirmatory factor analysis and exploratory structural equation modeling (ESEM) were used to determine factorial structure.

**Results:**

The ESEM model showed an acceptable fit and outperformed the confirmatory model. A 14‐item version of the ETMCQ was retained after examining item performance. Our findings also established criterion‐related validity for mistrust and credulity, an acceptable internal consistency for credulity, discriminant power for mistrust and credulity in detecting positive screens for borderline personality disorder, and measurement invariance across sexes.

**Conclusion:**

This study provides evidence for the cross‐cultural applicability of the ETMCQ. Nonetheless, the validity of the trust and internal consistency of the mistrust subscale require particular attention in future research.

## Graphical abstract

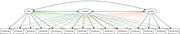

## 1 INTRODUCTION

Epistemic trust (ET) is an evolutionary advantage that allows us to learn and adapt efficiently by trusting in the conveyed knowledge as personally relevant, consequential, and generalizable (Fonagy et al., 2015). Humans are epistemically vigilant (Sperber et al., 2010), critically evaluating transmitted knowledge while trusting specific information. Accordingly, humans are argued to possess a filter mechanism for selecting knowledge (Csibra & Gergely, 2009; Fonagy et al., 2022): They exhibit sensitivity to specific cues indicating the importance and relevance of communications. These “ostensive cues” signal that addressees are acknowledged as agentive, unique individuals, making them receptive to transferred knowledge (Fonagy, Allison, et al., 2019).

Campbell et al. (2021) recently distinguished among three epistemic stances: ET, epistemic mistrust (EM), and epistemic credulity (EC). ET is the selective openness to social learning, which facilitates identifying and internalizing authentic self‐relevant knowledge. EM signifies a skeptical view of information sources, presupposing their lack of reliability or the possibility of concealed motives. On the other hand, EC manifests as an unwarranted, excessive trust in information sources, marked by a distinct lack of vigilance and discrimination (Campbell et al., 2021). Thus, EM may lead to active distancing from external influences and adopting a hypervigilant stance toward new information, whereas EC is often accompanied by a general lack of clarity about one's own position, rendering individuals vulnerable to manipulation and exploitation (Campbell et al., 2021).

Personality disorders, particularly borderline personality disorder (BPD), are increasingly understood as social‐communicative difficulties rather than inherent individual traits (Fonagy et al., 2015). This shift in perspective acknowledges that early attachment contexts, as well as the broader sociocultural environment, play a pivotal role in shaping relational strategies, as assumedly “maladaptive” attitudes and behaviors may serve the individuals’ well‐being in adverse milieux (Luyten et al., 2021). These attitudes and behaviors become truly maladaptive when they are upheld and generalized to the environments and relationships where trust may be warranted. Thus, micro and macro factors may contribute to EM and EC, where individuals struggle to balance self‐reliance and the need for social connection. Furthermore, mentalizing, or imagining mental states underlying behaviors, is an ostensive cue that fosters shared intentionality and initiates natural pedagogy. In essence, the addresser mentalizes the addressee's self‐narrative, and if this aligns with the addressee's perception of how the addresser views them, receptivity occurs (also called the we‐mode), preparing the addressee to internalize and generalize the knowledge (Fonagy et al., 2022; Fonagy, Luyten, et al., 2019). Hence, mentalizing deficits, which may have the same roots as EM and EC, could hinder an individual's ability to accurately imagine the mental states of the self and others in different ways, potentially leading to the avoidance of mental content, biased perceptions of reality, or an incoherent, scattered sense of self (Fonagy et al., 2022). In essence, the intertwined nature of attachment insecurities, mentalizing deficits, and epistemic stances creates a constellation of factors contributing to the development of the rigidity that characterizes BPD (Nolte et al., 2023).

To date, ET has primarily been the subject of theoretical developments. However, Campbell et al. (2021) recently developed and validated the Epistemic Trust, Mistrust, and Credulity Questionnaire (ETMCQ) as the first self‐report measure for adaptive and maladaptive forms of ET. The ETMCQ comprises 15 items using a scale ranging from 1 (*strongly agree*) to 7 (*strongly disagree*), and has been administered in a number of studies. Table 1 presents key findings of its psychometric properties across these studies. Potential challenges to the validity and reliability of the ETMCQ include factorial structure, criterion‐related validity, and internal consistency. First, Campbell et al. (2021) conducted an exploratory factor analysis primarily with 18 items, demonstrating a 3‐factor solution (with 6 items indicating each factor). However, the fit of this model was poor in confirmatory factor analysis (CFA), leading to the omission of one item from each factor and correlating residual variances of five item pairs to achieve a satisfactory fit. Although item 6 showed a low standardized factor loading in the CFA, this model was endorsed. On the other hand, Liotti, Milesi et al. (2023) conducted a principal components analysis with the 15‐item version. Due to the cross‐loading of item 11, the analyses were conducted again after its deletion, resulting in a three‐factor solution but with different factor compositions: In this structure, item 15 (*In the past, I have misjudged who to believe and been taken advantage of*), originally intended to indicate EC, was loaded on EM. Nonetheless, the resulting structure showed an acceptable fit in subsequent CFA. Second, despite inconclusive evidence regarding the criterion‐related validity of ET, most studies indicate nonsignificant or negligible associations between ET and relevant constructs (Benzi, Carone, et al., 2023; Benzi, Fontana, et al., 2023; Campbell et al., 2021; Kampling et al., 2022, 2024; Liotti, Milesi, et al., 2023; Tanzilli et al., 2022), with a minority substantiating its validity (Liotti, Fiorini Bincoletto, et al., 2023; Riedl et al., 2024; Riedl, Rothmund, et al., 2023), and a handful of investigations presenting mixed findings (Brauner et al., 2023; De Coninck et al., 2023; Hauschild et al., 2023; Parolin et al., 2023). Third, although good test–retest reliability was found for all three subscales, the majority of the findings demonstrated questionable internal consistency for EM (see Table 1).

**TABLE 1:** Key psychometric properties of the Epistemic Trust, Mistrust, and Credulity Questionnaire (ETMCQ) in previous studies.

Study	Sample characteristics	Factor analysis methods	Reliability
Campbell et al. (2021)	Community adults	EFA^a^ with Promax rotation CFA	Test–retest (*n* = 411) ICC_T_ = .85, ICC_M_ = .82, and ICC_C_ = .83 Internal consistency Sample 1 (discovery half; *n* = 250): *α*_T_ = .76, *α*_M_ = .72, *α*_C_ = .81 Sample 1 (confirmatory half; *n* = 250): *α*_T_ = .81, *α*_M_ = .70, *α*_C_ = .75 Sample 2 (*n* = 705): *α*_T_ = .69, *α*_M_ = .65, *α*_C_ = .81
Liotti, Milesi et al. (2023)^b^	Community adults	PCA with Varimax rotation CFA	*n* = 843; *α*_T_ = .73, *α*_M_ = .67, *α*_C_ = .73
Kampling et al. (2024)	Community; age ≥16 years	–	*n* = 2519; *α*_T_ = .81, *α*_M_ = .66, *α*_C_ = .81
Riedl et al. (2024)	Adult patients	–	*n* = 771; *α*_T_ = n/a, *α*_M_ = n/a, *α*_C_ = n/a
Riedl, Rothmund et al. (2023)	Adult patients	–	*n* = 249; *α*_T_ = .71, *α*_M_ = .57, *α*_C_ = .79
Brauner et al. (2023)	Community adults	–	*n* = 417; *α*_T_ = .73, *α*_M_ = .70, *α*_C_ = .77
Hauschild et al. (2023)	Community adults	–	*n* = 595; *α*_T_ = .78, *α*_M_ = .67, *α*_C_ = .81
De Coninck et al. (2023)	University students	–	*n* = 289; *α*_T_ = .70, *α*_M_ = .65, *α*_C_ = .81
Benzi, Fontana et al. (2023)	Community adults	–	*n* = 358; *ω*_T_ = .70, *ω*_M_ = .69, *ω*_C_ = .79
Damnjanović et al. (2023)	Community adults	–	Subsample 1 (health‐care providers; *n* = 219): *α*_T_ = .70, *α*_M_ = .66, *α*_C_ = .82 Subsample 2 (parents/caretakers; *n* = 263): *α*_T_ = .73, *α*_M_ = .56, *α*_C_ = .78 Subsample 3 (laypeople; *n* = 263): *α*_T_ = .70, *α*_M_ = .66, *α*_C_ = .77
Nimbi, Giovanardi, et al. (2023)	Community adults	–	*n* = 333; *α*_T_ = .90, *α*_M_ = n/a, *α*_C_ = .86
Nimbi, Baiocco et al. (2023)^c^	–	–	–
Fiorini Bincoletto et al. (2023)	Community adults	–	*n* = 301; *α*_T_ = n/a, *α*_M_ = n/a, *α*_C_ = n/a
Liotti, Fiorini Bincoletto, et al. (2023)	Nonclinical adolescents	–	*n* = 565; *α*_T_ = n/a, *α*_M_ = n/a, *α*_C_ = n/a^d^
Benzi, Carone, et al. (2023)	Emerging adults	–	*n* = 469; *α*_T_ = n/a, *α*_M_ = n/a, *α*_C_ = n/a
Parolin et al. (2023)	Nonclinical adolescents	–	*n* = 482; *α*_T_ = n/a, *α*_M_ = n/a, *α*_C_ = n/a
Tanzilli et al. (2022)	Community adults	–	*n* = 367; *α*_T_ = .70, *α*_M_ = .70, *α*_C_ = .76
Kampling et al. (2022)	Community; age ≥16 years	–	*n* = 2004; *α*_T_ = .81, *α*_M_ = .69, *α*_C_ = .80
Barnby et al. (2022)	Community adults^e^	–	*n* = 86; *α*_T_ = .72, *α*_M_ = .54, *α*_C_ = .72
Tanzer et al. (2021)	Community adults	–	Sample 1 (*n* = 705): *α*_T_ = .70, .65, *α*_C_ = .81 Sample 2 (*n* = 502): *α*_T_ = .73, *α*_M_ = .69, *α*_C_ = .70

*Notes*: *α*_T_ = trust *α*, *α*_M_ = mistrust *α*, *α*_C_ = credulity *α*, n/a = not available.

Abbreviation: CFA, confirmatory factor analysis.

^a^ Campbell et al. (2021) ambiguously stated, “…extracting principal factors…” which could be referring to either principal axis factoring or principal component analysis.

^b^ Factor composition of this study differs from others.

^c^ This study reused the data from Nimbi, Giovanardi et al. (2023).

^d^ Cronbach's alphas of the three subscales ranged from .67 to .72.

^e^ The results for the neurotypical sample are reported.

## 2 CURRENT STUDY

We aimed to evaluate the psychometric properties of the ETMCQ in the non‐Western, Educated, Industrialized, Rich, and Democratic (WEIRD; Henrich et al., 2010) context of Iran, as the limited inclusion of non‐WEIRD populations in social psychology research may hinder the development of a universally applicable definition of ET. Specifically, we intended to proceed with the following aims and hypotheses: applying exploratory structural equation modeling (ESEM) to test the factorial structure, with the hypothesis that our findings would replicate the theoretically established three‐dimensional model; evaluating the criterion‐related validity by examining associations among ET, EM, EC, and relevant constructs (i.e., mindfulness, perspective‐taking, and the umbrella concept of mentalizing; attachment insecurities; emotion dysregulation; and BPD traits), anticipating strong associations for EM and EC, but not for ET, in‐line with previous findings; examining the internal consistency of the three ETMCQ subscales, hypothesizing that EM would exhibit unacceptable results, whereas ET and EC would demonstrate satisfactory results; exploring the discriminant ability of the subscales in identifying individuals with positive and negative screens for BPD, expecting discriminant ability for EM and EC, but not for ET; testing the measurement invariance across sexes, guided by the hypothesis that sex invariance would be established due to the seemingly unbiased item formulations; and analyzing associations between sociodemographic variables and the three epistemic stances, with hypotheses aligned with previous research (for the three subscales, the presence of cross‐gender differences and insignificant or weak associations with age, level of education, marital status, and employment status; Benzi, Fontana, et al., 2023; Campbell et al., 2021; Liotti, Milesi, et al., 2023; Riedl, Rothmund, et al., 2023; Tanzer et al., 2021).

## 3 METHODS

### 3.1 Procedure and participants

One of the authors translated the ETMCQ into Persian, and a bilingual English/Persian translator back‐translated the items into English. The translated version was then compared to the original version by both authors, and existing discrepancies were corrected. Subsequently, the measure battery was created online using the Porsline platform (www.porsline.com), and the associated URL was shared via the most popular social media applications in Iran (i.e., Telegram, WhatsApp, and Instagram). The survey platform employed automated methods to confirm Iranian residency and safeguard against duplicate submissions.

A sample size of 10 times the number of free parameters (here, 72 for the 15‐item ESEM model) is required for SEM studies (Kline, 2023). Hence, a minimum sample size of 720 was determined. Moreover, attention checks and response time screening were used to address careless responding (Ward & Meade, 2023), which resulted in the deletion of 12 responses due to insufficient completion time. The data were collected in two sequential phases as part of a continuous project. Initially, a subsample of 497 participants (*M*_age_ = 29.73, SD_age_ = 10.29, range = 18–66) completed the initial battery. Consequently, we removed the perspective‐taking measures and added instruments for attachment insecurities and BPD (see Section 3.2). In the second phase, 409 participants (*M*_age_ = 33.72, SD_age_ = 10.57, range = 18–67) were recruited. These participants completed the updated battery. This sequential approach was used to enhance criterion‐related validity and discriminant ability assessment without adding to the participants’ burden and to maintain temporal continuity in data collection. The sociodemographic characteristics of the participants are presented in Table 2. The participants, in total, were relatively young and were representative of the mean age of 30 years in Iran. For the CFA/ESEM, internal consistency calculation, and measurement invariance testing of the ETMCQ, a combined dataset (*n* = 906) was used. Notably, the current dataset was partially used for a “Letter to the Editor” article conducted during the data collection process (Asgarizadeh, Hunjani, et al., 2023).

**TABLE 2:** Sociodemographic characteristics of participants.

		Subsample 1 (*n* = 497)		Subsample 2 (*n* = 409)	
Characteristics		Frequency	Percentage	Frequency	Percentage
Sex	Female	343	69	323	79
	Male	154	31	86	21
Age	18–24	187	37.6	86	21
	25–34	197	39.6	140	34.2
	35–44	58	11.7	109	26.7
	45–54	34	6.8	63	15.4
	55<	21	4.2	11	2.7
Relationship status	Single	262	52.7	182	44.5
	In a relationship	74	14.9	57	13.9
	Married	161	32.4	170	41.6
Level of education	Middle school	4	0.8	4	1
	High school	84	16.9	55	13.4
	Associate	17	3.4	11	2.7
	BSc	198	39.8	156	38.1
	MSc	149	30	162	39.6
	PhD/MD	45	9.1	21	5.1
Employment status	Employed	426	85.7	317	77.5
	Unemployed	71	14.3	92	22.5

### 3.2 Measures

The Reflective Functioning Questionnaire (RFQ; Fonagy et al., 2016) and the Mentalization Questionnaire (MZQ; Hausberg et al., 2012) were used to measure mentalizing deficits, as recent findings suggest their incremental utility (Raimondi et al., 2022). The RFQ employs a 7‐point scale ranging from *strongly disagree* to *strongly agree*. The initially proposed two‐factor structure has faced criticism in recent studies, and an alternative single‐factor model measuring uncertainty about mental states has been suggested (Woźniak‐Prus et al., 2022). The MZQ comprises 15 items rated on a 5‐point scale (*I disagree* to *I agree*). Rather than the four‐factor structure (Hausberg et al., 2012), its single‐factor solution has been widely substantiated (Ponti et al., 2019; Raimondi et al., 2022; Riedl, Kampling, et al., 2023). Contrary to Hausberg et al. (2012), we did not recode items to ensure that the scores reflect mentalizing deficits. Moreover, the findings support the criterion‐related validity and reliability of the single‐factor RFQ and the MZQ in Iran (Asgarizadeh, Sharp, et al., 2023). Both subsamples completed the RFQ and MZQ in this study.

The Iranian adaptation of the Revised Adult Attachment Scale (RAAS; Asgarizadeh, Pakdaman, et al., 2023; Collins, 1996) consists of 12 items, rated on a 5‐point scale ranging from *Not at all characteristic of me* to *Very characteristic of me*. It assesses attachment insecurities through two 6‐item subscales of anxiety and avoidance. Elevated scores on these subscales indicate greater levels of attachment insecurities. Previous studies have corroborated the psychometric properties of the adapted RAAS (Asgarizadeh, Pakdaman, et al., 2023). The RAAS was administered only in the second subsample.

The Mindful Attention Awareness Scale (MAAS; Brown & Ryan, 2003) measures individuals’ ability to maintain present‐moment attention and awareness. It comprises 15 items, and respondents rate each item on a scale ranging from 1 (*almost always*) to 6 (*almost never*). Higher MAAS scores signify a greater capacity to embrace the present moment. A single‐factor structure has been supported for the Persian version of the MAAS (Nooripour et al., 2023). Both subsamples responded to the MAAS.

The perspective‐taking subscale of the interpersonal reactivity index (IRI‐PT; Davis, 1983) was employed to capture cognitive empathy. It consists of seven items rated on a 5‐point scale (*does not describe me well* to *describes me well*), two of which are reversely coded. In Iranian samples, the IRI‐PT is unidimensionally structured and is the best indicator of cognitive empathy among the IRI subscales (Yaghoubi Jami & Wind, 2022). Only the first subsample completed the IRI‐PT.

The Iranian adaptation of the Difficulties in Emotion Regulation Scale—Short Form (DERS—SF; Asgarizadeh, Mazidi et al., in press; Kaufman et al., 2016) was used to measure emotion dysregulation, which consists of 15 items (excluding the awareness subscale). Respondents rate items using a 5‐point scale, ranging from *almost never* to *almost always*, where higher scores reflect greater challenges in regulating emotions. The psychometric properties of this version were strongly corroborated in a sample of Iranian adults (Asgarizadeh, Mazidi, et al., in press). The DERS—SF was administered to both subsamples.

The McLean Screening Instrument for BPD (MSI‐BPD; Zanarini et al., 2003) was employed to measure BPD traits. Comprising 10 binarily responded items, the MSI‐BPD generates scores indicative of the frequency of BPD symptoms, with higher scores denoting a greater presence. Furthermore, the criterion‐related validity and reliability of its Persian translation have been supported in previous research (Mousavi Asl et al., 2020). In this study, only the second subsample completed the MSI‐BPD.

### 3.3 Data analysis

The CFA model suggested by Campbell et al. (2021) was compared against its ESEM counterpart with robust maximum likelihood estimation—which controls for multivariate non‐normality—and target rotation (Morin, 2023). ESEM effectively combines the strengths of CFA and exploratory factor analysis while addressing their respective limitations (Asparouhov & Muthén, 2009). ESEM recognizes that data often have intricate structures beyond simple factor structures and models these relationships realistically. In other words, it offers greater accuracy and flexibility than CFA by not imposing constraints on cross‐loadings, delivering more precise estimates of latent variable correlations, and offering a less biased representation of data (Marsh et al., 2014). Due to the previous reasons, this approach has become a preferred method for examining model fit in multidimensional measures (Morin, 2023). Notably, target rotation offers a confirmatory approach by adjusting factor structures to fit predefined correlation matrices. Unlike traditional rotation methods, it focuses on specific correlations between factors and is preferred for strong theoretical models, allowing for freely estimated main loadings while setting cross‐loadings to be close to zero (Asparouhov & Muthén, 2009; Marsh et al., 2014).

Three indices were used to assess model fit: the comparative fit index (CFI), the root mean square error of approximation (RMSEA), and the standardized root mean square residual (SRMR) (Kline, 2023). The following values indicated acceptable fit: CFI ≥ .90, RMSEA, and SRMR ≤ .10 (Meyers et al., 2017). A minimum standardized factor loading of.30 was considered acceptable (Brown, 2015). Furthermore, item uniquenesses (i.e., residual error variances; .10 < *δ* < .90), standard errors, and reliability indicators (corrected item‐total correlation [CITC] > .30, Cronbach's alpha and multidimensional correlated factors reliability >.70; Cho, 2022; van Zyl & ten Klooster, 2022) were examined to evaluate measurement quality (Asparouhov & Muthén, 2009).

Criterion‐related validity was assessed by examining Pearson correlation coefficients with instruments measuring related constructs. Correlation coefficients were categorized as small (*r* = .10), medium (*r* = .20), and large (*r* = .30) (Funder & Ozer, 2019; Gignac & Szodorai, 2016). Moreover, to investigate the ability of the ETMCQ subscales to distinguish individuals with positive and negative screens for BPD diagnosis (i.e., MSI‐BPD scores ≥7 and <7, respectively), receiver operating characteristic curves were plotted, and area under curve (AUC) values were estimated. A significant AUC value greater than .70 indicates good discriminant ability (Šimundić, 2009). All analyses were performed using Mplus v8.3, IBM SPSS v27, and MedCalc v20.104. The Mplus syntax for the ESEM and invariance testing was generated using De Beer and van Zyl (2019) and De Beer and Morin (2022) tools, respectively.

Additionally, the best fitting model was chosen to test measurement invariance across sexes. Configural (equal structure), metric (equal loadings), scalar (equal intercepts), and residual (equal residuals) invariances were progressively tested. For all model comparisons, the following criteria indicated difference: significance of Satorra–Bentler scaled chi‐square difference test and a ΔCFI ≥ −.01 coupled with either a ΔRMSEA ≥ .015 or a ΔSRMR ≥ .010 (Chen, 2007; Hair et al., 2019). Moreover, to compare the CFA and ESEM models, the Akaike information criterion and the Bayesian information criterion were utilized, with lower values indicating a better fit. Provided that the metric invariance was upheld, mean scores of the subscales were compared across sexes using *t*‐tests.

## 4 RESULTS

Our results indicate a poor fit for the CFA but an acceptable fit for the ESEM model (Table 3). The ESEM model also demonstrated a significantly better fit than its CFA counterpart. Moreover, the ESEM model yielded lower absolute factor correlations than the CFA model (mean *r* = .18, compared to mean *r* = .28), indicating better factor differentiation (Marsh et al., 2014). Nonetheless, the CITC for item 14 was questionable, and in the ESEM model, item 14 (*I don't usually act on advice that I get from others even when I think it's probably sound*) loaded poorly on EM, its proposed factor. Moreover, compared to item 14, two other items intended for ET and EC (items 1 and 15) exhibited stronger factor loadings on EM. Hence, we reconducted the ESEM with the 14‐item structure (Table 4).

**TABLE 3:** Fit indices for and comparison between confirmatory factor analysis (CFA) and exploratory structural equation modeling (ESEM) models (original structure).

Model	*χ* ^2^ (df)	CFI	RMSEA [90% CI]	SRMR	AIC	BIC	Decision
CFA	432.92 (87)	.815	.066 [.060–.073]^***^	.063	49,020.76	49,251.59	Reject
ESEM	198.71 (63)	.923	.049 [.041–.056]^ns^	.030	48,788.26	49,134.51	Accept
**Comparison**	**S–B‐scaled *χ* ^2^ (Δdf)**	**ΔCFI**	**ΔRMSEA**	**ΔSRMR**	**ΔAIC**	**ΔBIC**	**Difference**
	233.21 (24)^***^	.108	−.017	−.033	−232.5	−117.08	Yes

*Notes*: S–B = Satorra–Bentler. ns = nonsignificant (*p* > .05), ^***^ *p *< .001.

Abbreviations: AIC, Akaike information criterion; BIC, Bayesian information criterion; CFI, comparative fit index; RMSEA, root mean square error of approximation; SRMR, standardized root mean square residual.

**TABLE 4:** Item analysis and exploratory structural equation modeling (ESEM) estimates for the original and 14‐item Epistemic Trust, Mistrust, and Credulity Questionnaire (ETMCQ).

							15‐Item (original) version								14‐Item version (item 14 excluded)							
							Trust		Mistrust		Credulity				Trust		Mistrust		Credulity			
Factor	Item	*M*	SD	SK	KU	CITC	*λ*	SE	*λ*	SE	*λ*	SE	*δ*	*R* ^2^	*λ*	SE	*λ*	SE	λ	SE	*δ*	*R* ^2^
Trust	1	4.83	1.66	−.57	−.40	.40	**.49**	.04	−.29	.04	.13	.03	.63	.37	**.50**	.04	−.28	.04	.11	.03	.63	.37
	2	5.50	1.31	−.90	.85	.47	**.56**	.04	.03	.04	.03	.03	.69	.31	**.57**	.04	.03	.04	.02	.03	.68	.32
	7	5.20	1.39	−.79	.53	.38	**.50**	.05	.11	.05	.08	.04	.74	.26	**.50**	.05	.11	.05	.07	.04	.74	.26
	8	5.78	1.16	−.98	1.29	.44	**.59**	.05	.08	.04	−.08	.03	.66	.34	**.59**	.05	.09	.04	−.10	.03	.67	.33
	13	5.45	1.35	−.93	.84	.43	**.56**	.04	.05	.04	−.01	.04	.69	.31	**.56**	.04	.05	.04	−.02	.04	.69	.31
Mistrust	3	4.69	1.55	−.21	−.59	.32	−.13	.04	**.42**	.05	−.05	.04	.80	.20	−.14	.04	**.41**	.05	−.03	.04	.80	.20
	4	4.60	1.73	−.27	−.88	.38	−.11	.04	**.50**	.05	.18	.04	.64	.36	−.13	.04	**.49**	.05	.21	.04	.63	.37
	9	4.97	1.52	−.44	−.35	.35	.10	.04	**.55**	.05	−.07	.03	.72	.28	.08	.04	**.55**	.05	−.05	.03	.72	.28
	10	4.65	1.52	−.21	−.56	.34	.16	.04	**.49**	.05	.03	.04	.75	.25	.14	.04	**.48**	.05	.05	.04	.76	.25
	14	3.19	1.82	.56	−.32	.27	−.19	.04	.26	.05	.23	.04	.80	.20	–	–	–	–	–	–	–	–
Credulity	5	2.99	1.90	.64	−.76	.57	.01	.03	.01	.04	**.70**	.04	.51	.49	−.01	.03	−.01	.04	**.70**	.04	.51	.49
	6	2.89	1.69	.66	−.45	.48	.09	.04	−.05	.04	**.62**	.04	.62	.38	.08	.04	−.06	.04	**.62**	.04	.62	.38
	11	3.40	1.68	.44	−.61	.53	−.03	.04	.19	.04	**.53**	.04	.62	.38	−.05	.04	.18	.04	**.54**	.04	.62	.38
	12	3.19	1.82	.48	−.85	.60	.04	.03	−.11	.03	**.78**	.03	.42	.58	.03	.03	−.12	.03	**.78**	.03	.42	.58
	15	4.46	1.94	−.20	−1.13	.35	−.01	.04	.27	.04	**.32**	.04	.77	.23	−.02	.04	.25	.04	**.33**	.04	.78	.22

*Notes*: Standardized loadings >.30 are in bold. *λ* = standardized factor loading; *δ* = item uniqueness (residual variances).

Abbreviations: *M*, mean; SD, standard deviation; SK, skewness; KU, kurtosis; CITC, corrected item‐total correlation; SE, standard error.

**FIGURE 1:**
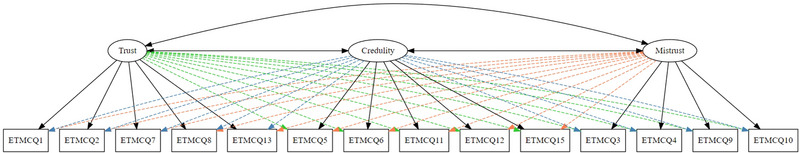
The exploratory structural equation modeling (ESEM) model for the 14‐item Epistemic Trust, Mistrust, and Credulity Questionnaire (ETMCQ). Solid black and dashed colored lines represent main loadings and cross‐loadings, respectively.

The 14‐item model demonstrated acceptable fit (*χ* ^2^ (52) = 189.56, *p* < .001; CFI = .922; RMSEA = .054, 90% CI = [.046–.062]; SRMR = .031), standardized loadings (*λ*s = .33–.78, *M*_λ_ = .54), item uniquenesses (.42 < *δ* < .80), and CITC values (>.32), with no cross‐loadings (*λ* < .28). Although item 15 showed proximate loadings on EC (.33) and EM (.25), as it conceptually relates to EC and as its loading on EM does not cross the acceptable threshold of .30, we retained it as an indicator of EC. However, acceptable multidimensional internal consistency coefficients were only found for EC (*α* = .74, *ρ* = .75), unlike EM (*α* = .56, *ρ* = .56) and ET (*α* = .66, *ρ* = .67). Given that the same problem existed for the 5‐item EM (*α* = .58, *ρ* = .58), the 14‐item structure was retained for all further analyses (Figure 1).

**FIGURE 2:**
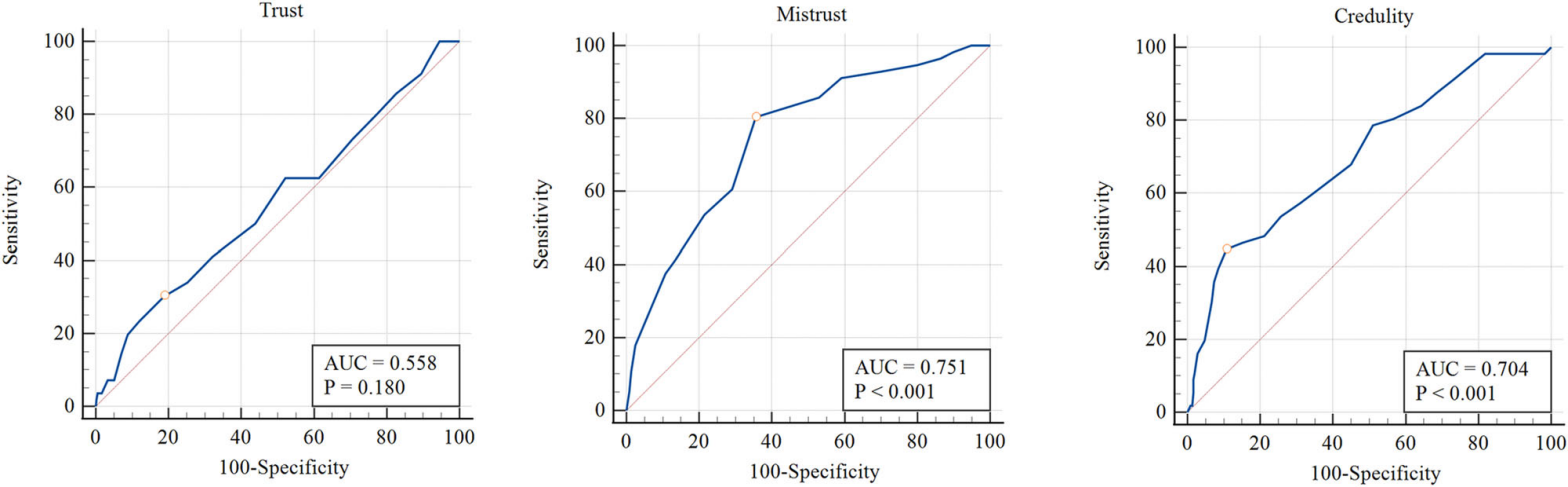
Receiver operating characteristic (ROC) curve graphs examining the ability of Epistemic trust (ET), epistemic mistrust (EM), and epistemic credulity (EC) in discriminating individuals with positive screen (i.e., MSI‐BPD score ≥7) and negative screen (i.e., MSI‐BPD score <7) of BPD diagnosis. MSI‐BPD, McLean Screening Instrument for borderline personality disorder.

Tables 5 and 6 present descriptive statistics and Pearson correlation coefficients for the first and second subsamples, respectively (total‐sample and sex‐specific descriptive statistics for the ETMCQ subscales are available in Table S1). The correlations between EM, EC, and related constructs were statistically significant and mostly large in magnitude (EM: |*r*| = .13–.48, *M*_|_ *_r_*_|_ = .33; EC: |*r*| = .27–.53, *M*_|_ *_r_*_|_ = .44). Compared to EM, EC demonstrated stronger correlations with mentalizing‐related constructs and attachment anxiety and weaker correlations with attachment avoidance. ET, on the other hand, showed weak or negligible correlations (|*r*| = .01–.17, *M*_|_ *_r_*_|_ = .06). Furthermore, individuals with a positive screen (*n* = 56, 13.69%) and negative screen (*n* = 353, 86.31%) for BPD were discriminated by the scores of EM (odds ratio = 2.746, Nagelkerke's *R* ^2^ = .169, AUC = .751, 95% CI = .706–.792, SE = .034, *p* < .001) and EC (odds ratio = 1.828, Nagelkerke's *R* ^2^ = .124, AUC = .704, 95% CI = .657–.748, SE = .039, *p* < .001), but not ET (odds ratio = .768, Nagelkerke's *R* ^2^ = .013, AUC = .558, 95% CI = .509–.607, SE = .043, *p* > .05) (Figure 2). According to Youden's index (.447), EM mean scores >4.75 categorized individuals with a sensitivity of 80.36 and a specificity of 64.31. For EC, Youden's index (.339) suggested that mean scores > 4.4 categorized individuals with a sensitivity of 44.64 and a specificity of 89.24.

**TABLE 5:** Descriptive statistics and Pearson correlation coefficients for the first subsample (*n* = 497).

	Measure	Subscale	*M*	SD	*α*	1	2	3	4	5	6	7
1	ETMCQ	Trust	5.3	.89	.67	1						
2		Mistrust	4.72	1.08	.49	−.10^*^	1					
3		Credulity	3.45	1.27	.73	.15^**^	.26^**^	1				
4	RFQ	–	3.64	1.25	.82	.03	.35^**^	.49^**^	1			
5	MZQ	–	2.84	.72	.83	−.02	.48^**^	.45^**^	.62^**^	1		
6	MAAS	–	4.32	.75	.89	−.04	−.29^**^	−.43^**^	−.59^**^	−.54^**^	1	
7	DERS‐SF	–	2.48	.80	.95	.03	.34^**^	.44^**^	.74^**^	.65^**^	−.62^**^	1
8	IRI	Perspective taking	3.59	.56	.76	.17^*^	−.13^**^	−.27^**^	−.45^**^	−.34^**^	.35^**^	−.36^**^

Abbreviations: ETMCQ, Epistemic Trust, Mistrust, and Credulity Questionnaire; RFQ, Reflective Functioning Questionnaire; MZQ, Mentalization Questionnaire; MAAS, Mindfulness Attention Awareness Scale; IRI, interpersonal reactivity index; DERS‐SF, Difficulties in Emotion Regulation Scale—Short Form.

^*^ *p* < .05, ^**^ *p* < .01.

**TABLE 6:** Descriptive statistics and Pearson correlation coefficients for the second subsample (*n* = 409).

	Measure	Subscale	*M*	SD	*α*	1	2	3	4	5	6	7	8	9
1	ETMCQ	Trust	5.39	.92	.67	1								
2		Mistrust	4.74	.99	.49	−.13**	1							
3		Credulity	3.31	1.26	.73	.05	.30**	1						
4	RFQ	–	3.60	1.32	.82	.08	.29**	.53**	1					
5	MZQ	–	2.83	.74	.83	−.03	.42**	.49**	.64**	1				
6	RAAS	Anxiety	2.83	1.04	.86	.01	.27**	.48**	.57**	.65**	1			
7		Avoidance	2.85	.91	.80	−.13*	.43**	.33**	.47**	.61**	.62**	1		
8	MAAS	–	4.42	.79	.89	.04	−.26**	−.43**	−.50**	−.57**	−.49**	−.44**	1	
9	DERS−SF	–	2.32	.87	.95	.03	.29**	.44**	.66**	.63**	.63**	.54**	−.63**	1
10	MSI−BPD	–	.35	.25	.76	−.11*	.39**	.47**	.60**	.57**	.56**	.50**	−.47**	.56**

Abbreviations: DERS‐SF, Difficulties in Emotion Regulation Scale—Short Form; ETMCQ, Epistemic Trust, Mistrust, and Credulity Questionnaire; MAAS, Mindfulness Attention Awareness Scale; MSI‐BPD, McLean Screening Instrument for Borderline Personality Disorder; MZQ, Mentalization Questionnaire; RAAS, Revised Adult Attachment Scale; RFQ, Reflective Functioning Questionnaire.

*p < .05, ^**^ *p* < .01.

Stepwise measurement invariance testing supported configural invariance and demonstrated no significant changes in model fit indices for metric, scalar, and residual invariances (Table 7). Subsequently, sex differences were found in the mean scores of ET (*M*_Females_ = 5.41, SD_Females_ = .91, *M*_Males_ = 5.19, SD_Males_ = .87, *t*_(904)_ = 3.22, *p* < .01, Cohen's *d* = .24) and EM (*M*_Females_ = 4.65, SD_Females_ = 1.03, *M*_Males_ = 4.95, SD_Males_ = 1.03, *t*_(904)_ = −3.91, *p* < .001, Cohen's *d* = −.29), but not EC (*M*_Females_ = 3.34, SD_Females_ = 1.25, *M*_Males_ = 3.51, SD_Males_ = 1.29, *t*_(904)_ = −1.84, *p* > .05). Regarding other sociodemographic variables, the Spearman correlation coefficients showed mostly small coefficients for ET (age: *r*s = .07, *p* < .05; marital status: *r*s = .09, *p* < .01; education level: *r*s = −.03, *p* > .05), EM (age: *r*s = −.12, *p* < .01; marital status: *r*s = −.11, *p* < .01; education level: *r*s = −.20, *p* < .01), and EC (age: *r*s = −.05, *p* > .05; marital status: *r*s = −.06, *p* > .05; education level: *r*s = −.10, *p* < .01). Additionally, there were no differences between employed and unemployed participants regarding ET, EM, and EC scores (*p*s > .05).

**TABLE 7:** Measurement invariance for the 14‐item exploratory structural equation modeling (ESEM) model across sexes.

Model	*χ* ^2^ (df)	CFI	RMSEA [90% CI]	SRMR	S–B scaled *χ* ^2^ (Δdf)	ΔCFI	ΔRMSEA	ΔSRMR	Decision
Configural	266.61 (104)	.910	.059 [.050–.068]	.035	–	–	–	–	Accept
Metric	294.32 (137)	.913	.05 [.042–.058]	.042	31.18 (33)^ns^	.003	−.009	.007	Accept
Scalar	313.23 (148)	.908	.05 [.042–.057]	.044	18.18 (11)^ns^	−.005	0	.002	Accept
Residual	334.85 (162)	.904	.049 [.041–.056]	.055	22.23 (14)^ns^	−.004	−.001	.011	Accept

*Notes*: S–B = Satorra–Bentler. ns = nonsignificant (*p* > .05).

Abbreviations: CFI, comparative fit index; RMSEA, root mean square error of approximation; SRMR, standardized root mean square residual.

## 5 DISCUSSION

We examined the psychometric properties of the ETMCQ in the non‐WEIRD context of Iran. Our findings suggested an alternative factor structure, supported criterion‐related validity for EM and EC (although not ET), yielded acceptable reliability for EC (although not for ET and EM), demonstrated the discriminant power of EM and EC in detecting positive screen cases for BPD, and established measurement invariance across sexes.

Consistent with previous findings (Campbell et al., 2021; Liotti, Milesi, et al., 2023), this study supported the three‐factor structure of the ETMCQ. However, we found weak factor loading and CITC for item 14, as well as low internal consistency for EM and ET. As previous studies unanimously found acceptable internal consistency for ET (Table 1) and the coefficients in the current study are bordering on the thresholds, this finding is likely to be sample‐specific. On the other hand, similar observations of low internal consistency for EM are evident in the majority of previous studies (Table 1). As internal consistency coefficients are a function of inter‐item correlations (Meyers et al., 2017), it may be inferred that the items tap into separate aspects of EM. For instance, the focus of item 14 on behavioral responses, as opposed to the predominantly attitudinal nature of other EM items, could have led to this outcome. Additionally, the item's presumed soundness of advice creates a conceptual mismatch with the trait‐like skepticism of mistrustful individuals. Future research should delve into the conceptual coherence of EM items and investigate, through a qualitative lens, whether respondents fully grasp the rather abstract statements of the ETMCQ. This could inform potential revisions to simplify item wording or to add additional items.

Unlike ET, which demonstrated negligible associations, we found strong links among EM, EC, mentalizing and related constructs, attachment insecurities, and BPD traits. Accordingly, EM and EC discriminated individuals with positive and negative screen for BPD diagnosis, whereas ET did not. A study partly using the current dataset also demonstrated that, in predicting mentalizing and related constructs, ET has no‐to‐little incremental validity over and above EM and EC (Asgarizadeh, Hunjani, et al., 2023). These findings are consistent with a large body of evidence (Benzi, Carone, et al., 2023; Benzi, Fontana, et al., 2023; Campbell et al., 2021; Kampling et al., 2022, 2024; Liotti, Milesi, et al., 2023; Tanzilli et al., 2022). One possible explanation for this is that both extremely high and extremely low scores on ET may indicate epistemic disruptions. In other words, the relationship between ET and other constructs may be nonlinear. Furthermore, in the two abovementioned studies in which clinical samples were recruited, ET scores significantly improved over the course of psychosomatic rehabilitation, albeit with small effect sizes (Riedl et al., 2024; Riedl, Rothmund, et al., 2023). Thus, future studies may investigate the nonlinearity of the ET associations in nonclinical samples, administer the ETMCQ to individuals with a wider range of diagnoses, and, in the case of replicating our findings, examine the potential redundancy of ET in nonclinical samples.

Regarding the criterion‐related validity of the ETMCQ, the magnitude of correlations was generally similar to that of previous studies (Benzi, Carone, et al., 2023; Brauner et al., 2023; Campbell et al., 2021; Hauschild et al., 2023; Liotti, Milesi, et al., 2023; Parolin et al., 2023): EM and EC were strongly associated with mentalizing capacity, attachment insecurities, emotion dysregulation, and personality pathology. However, contrary to Liotti, Milesi et al. (2023), EC was more strongly related to uncertainty (i.e., RFQ score) than EM. Similarly, compared to EM, EC was more strongly associated with specific aspects of mentalizing, namely mindfulness and perspective‐taking. It has been suggested that self‐reported measures of mentalizing assess “perceived” mentalizing capacity (Wendt et al., 2023). Thus, stronger links for EC are plausible: Past experiences of credulous trust may diminish self‐confidence in mind‐reading ability. In turn, decreased confidence in one's mentalizing capacity makes the individual susceptible to excessively trusting others’ judgments and being subject to exploitation.

On attachment insecurities, our findings corresponded with those of Campbell et al. (2021), revealing that EM is more strongly related to avoidance and that EC is more strongly related to anxiety rather than vice versa. Luyten et al. (2021) congruently argued that avoidance is an adaptation to environments promoting self‐sufficiency, whereas anxiety may best serve in unpredictable milieux. Thus, avoidance may engender EM and closeness of the epistemic channel, whereas anxiety helps keep the channel open at the expense of self‐agency (Campbell et al., 2021). Lastly, for emotion dysregulation, the strong associations with maladaptive epistemic stances are theoretically underpinned: EM thwarts benefiting from co‐regulation with significant others and increases the chance of negatively interpreting neutral interpersonal stimuli, whereas EC makes the individual susceptible to accepting negative information about themselves and the world. These stances may also relate to emotion dysregulation through mentalizing deficits (Fonagy & Allison, 2023).

For the first time, our findings supported configural, metric, scalar, and residual invariances for the ETMCQ across females and males, indicating that its fundamental structure remains consistent, that the links between observed items and latent constructs (i.e., factor loadings) are equivalent, that response scales are interpreted uniformly, and that any unique item‐specific variability is comparable. As these invariances corroborated the suitability of the ETMCQ for meaningful cross‐sex contrasts, we compared mean scores, revealing that females scored higher on ET and lower on EM. This finding is partially congruent with Campbell et al. (2021), who found similar patterns for ET and EM but also significant differences for EC; partially congruent with Benzi, Fontana et al. (2023) and Riedl, Rothmund et al. (2023), who found similar patterns but only for ET; and inconsistent with Liotti, Milesi et al. (2023), who found no cross‐gender differences for the subscales. Cross‐cultural differences in interpersonal trust are well established (e.g., Luo et al., 2023; Mealy et al., 2015), but cultural variations in ET should be further explored.

This study has several limitations. First, no other self‐report measure of ET was developed when conducting this study. Recently, Knapen et al. (2024) introduced another measure for ET, which could be used in future research to examine the convergent validity of the ETMCQ. Additionally, relying solely on self‐report measures may introduce response biases and common method effects, potentially inflating the strength of reported associations. Future research could employ hetero‐method designs, such as combining the ETMCQ with experimental measures (Schröder‐Pfeifer et al., 2022) to mitigate this issue. Moreover, we found questionable internal consistencies for ET and EM, potentially threatening the validity of our findings. Furthermore, the subsamples were unbalanced with respect to sex and marital status, potentially limiting the generalizability of our findings. Finally, caution should also be exercised when generalizing these findings to individuals with clinical diagnoses, since the study samples consisted of community‐dwelling participants.

## AUTHOR CONTRIBUTIONS

**Ahmad Asgarizadeh**: Conceptualization; methodology; formal analysis; investigation; resources; writing—original draft; visualization; writing—review and editing; data curation. **Saeed Ghanbari**: Writing—review and editing; resources.

## CONFLICT OF INTEREST STATEMENT

The authors have no conflicts of interest to disclose.

## FUNDING INFORMATION

No funds, grants, or other support were received.

### PEER REVIEW

The peer review history for this article is available at https://publons.com/publon/10.1002/brb3.3455.

## Supporting information


**Table S1** Total‐sample and sex‐specific descriptive statistics for the ETMCQ subscales.

## Data Availability

The data supporting the findings of this study are available from the corresponding author without undue reservation.
