# Supplementary material for: Iranian adaptation of the Epistemic Trust, Mistrust, and Credulity Questionnaire (ETMCQ): Validity, reliability, discriminant ability, and sex invariance
Source: Brain Behav. 2024 Mar 7;14(3):e3455. doi: 10.1002/brb3.3455 (PMC10918607; doi:10.1002/brb3.3455)
Supplement: Supplementary file 1 — Table S1 Total‐sample and sex‐specific descriptive statistics for the ETMCQ subscales. [file BRB3-14-e3455-s001.docx]

**Supplementary Materials**

| **Table S1.** Total-sample and sex-specific descriptive statistics for the ETMCQ subscales. | | | | | | | | | | | | |
| --- | --- | --- | --- | --- | --- | --- | --- | --- | --- | --- | --- | --- |
|  | Total sample (n = 906) | | | | Females (n = 666) | | | | Males (n = 240) | | | |
| Subscales | M | SD | SK | KU | M | SD | SK | KU | M | SD | SK | KU |
| Trust | 5.35 | .90 | -.48 | .58 | 5.41 | .91 | -.62 | .98 | 5.19 | .87 | -.11 | -.21 |
| Mistrust | 4.73 | 1.04 | -.21 | .05 | 4.65 | 1.03 | -.12 | -.07 | 4.95 | 1.03 | -.48 | .74 |
| Credulity | 3.39 | 1.27 | .46 | -.02 | 3.34 | 1.25 | .46 | .04 | 3.51 | 1.29 | .46 | -.16 |
| *Notes.* M = Mean, SD = Standard Deviation, SK = Skewness, KU = Kurtosis. | | | | | | | | | | | | |
